# Feline coronavirus influences the biogenesis and composition of extracellular vesicles derived from CRFK cells

**DOI:** 10.3389/fvets.2024.1388438

**Published:** 2024-07-18

**Authors:** Sandani V. T. Wijerathne, Rachana Pandit, Ayodeji O. Ipinmoroti, Brennetta J. Crenshaw, Qiana L. Matthews

**Affiliations:** ^1^Microbiology Program, Alabama State University, Montgomery, AL, United States; ^2^Department of Biological Sciences, College of Science, Technology, Engineering, and Mathematics, Alabama State University, Montgomery, AL, United States

**Keywords:** feline coronavirus, pandemic, CRFK cells, extracellular vesicles, exosomes, immunomodulation, biogenesis

## Abstract

**Introduction:**

Coronavirus (CoV) has become a public health crisis that causes numerous illnesses in humans and certain animals. Studies have identified the small, lipid-bound structures called extracellular vesicles (EVs) as the mechanism through which viruses can enter host cells, spread, and evade the host’s immune defenses. EVs are able to package and carry numerous viral compounds, including proteins, genetic substances, lipids, and receptor proteins. We proposed that the coronavirus could alter EV production and content, as well as influence EV biogenesis and composition in host cells.

**Methods:**

In the current research, Crandell-Rees feline kidney (CRFK) cells were infected with feline coronavirus (FCoV) in an exosome-free media at a multiplicity of infection (MOI) of 2,500 infectious units (IFU) at 48 h and 72 h time points. Cell viability was analyzed and found to be significantly decreased by 9% (48 h) and 15% (72 h) due to FCoV infection. EVs were isolated by ultracentrifugation, and the surface morphology of isolated EVs was analyzed via Scanning Electron Microscope (SEM).

**Results:**

NanoSight particle tracking analysis (NTA) confirmed that the mean particle sizes of control EVs were 131.9 nm and 126.6 nm, while FCoV infected-derived EVs were 143.4 nm and 120.9 nm at 48 and 72 h, respectively. Total DNA, RNA, and protein levels were determined in isolated EVs at both incubation time points; however, total protein was significantly increased at 48 h. Expression of specific protein markers such as TMPRSS2, ACE2, Alix, TSG101, CDs (29, 47, 63), TLRs (3, 6, 7), TNF-α, and others were altered in infection-derived EVs when compared to control-derived EVs after FCoV infection.

**Discussion:**

Our findings suggested that FCoV infection could alter the EV production and composition in host cells, which affects the infection progression and disease evolution. One purpose of studying EVs in various animal coronaviruses that are in close contact with humans is to provide significant information about disease development, transmission, and adaptation. Hence, this study suggests that EVs could provide diagnostic and therapeutic applications in animal CoVs, and such understanding could provide information to prevent future coronavirus outbreaks.

## 1 Introduction

Coronaviruses (CoVs) are enveloped viruses with positive-single-stranded RNA, members of the *Nidovirales* order, and the *Coronaviridae* family (1, 2). Several reports strongly suggest that coronavirus has a zoonotic origin from bats. In SARS-CoV-2, angiotensin-converting enzyme 2 (ACE2) is used as the cellular entry receptor for virus entry into hosts (3–5). Numerous CoVs affect severe illnesses in animals, including canine, feline, dromedary camels, porcine, bovine, bird, and murine hepatitis virus (6). To gain insight into the effects of human CoV and animal CoVs, and potentially slow the next CoV pandemic, it is beneficial to perform more CoV research, including animal CoV research. Numerous studies have proved that canine–feline recombinant alphacoronavirus can cause diseases in humans, such as pneumonia and acute respiratory symptoms (7–9). Feline CoV is a highly contagious single-stranded RNA virus that infects cats and was first reported in 1960 (10, 11). It is primarily found in the gastrointestinal tract of cats, and it mainly causes asymptomatic infections (12). FCoV contains two main biotypes of FCoV: enteric FCoV (FECV) and feline infectious peritonitis (FIP) (13). FIP is a more severe and fatal form of systemic disease in young felines (14). FCoV is spread via the feces of infected cats, and the FCoV can remain contagious in dry fecal debris for up to 7 weeks (10). Previous research has demonstrated that there is no effective treatment for FCoV (15). Severe acute respiratory syndrome coronavirus-2 (SARS-CoV-2) and FCoV are able to respond to similar anti-inflammatory or antiviral compounds, and research on FCoV infection in cats can improve the knowledge concerning host-virus interaction (16). The first stage of CoV infection constitutes the binding of the coronavirus’ crown shape spike (S) protein to the cellular entry receptors (17). These entry receptors are mainly recognized by numerous CoVs, which involve human aminopeptidase N, ACE2, and dipeptidyl peptidase 4 (DPP4) (1, 18).

Extracellular vesicles (EVs), mainly the exosomes, are extensively researched in viral infections (8, 19). They are released at the early initial and final stages of the steadily increasing viral infection. EVs are able to make cells more susceptible to virus infections by conveying virus-specific host receptors to the cells (20, 21). EVs originate from cells and are enveloped by membranes that are secreted by most cell types into the extracellular space (22). They perform a significant role in facilitating cell signaling and are able to transport different types of molecules (23, 24). They are associated with various physiological and pathological processes, including immune control, immune reaction, cell differentiation, and cancer (25). EVs are nanoparticles of a range of sizes and shapes, including surface receptors, ribonucleic acids, membrane and soluble proteins, and lipids (26). They can be secreted and detected from biological fluids such as blood, urinary samples, breast milk, plasma, saliva, semen, ascitic fluid, spinal fluid, and bronchoalveolar lavage (27–31). Microvesicles (MVs), apoptotic bodies, and exosomes are the three main subtypes of EVs, and they are categorized based on their origin, composition, biological functions, dimensions, and emission pathways (24, 32, 33). The diameter of microvesicles can vary, typically ranging from 50 to 1,000+ nanometers, and they are secreted into extracellular space through the processes of outward budding and pinching of the cell membrane (34). Apoptotic bodies are the largest-sized EVs, generally spanning from 50 to 5,000 nanometers in diameter. These are formed from apoptosis and play significant roles in inflammation and immune responses (35). Exosomes are the smallest EVs, ranging from 30 to 200 nm (36). Mainly, exosomes are discharged into interstitial space by the multivesicular bodies (MVBs) fusing with the cytoplasmic membrane and are able to transport cell-specific cargos such as proteins, lipids, and genetic materials (36, 37). Exosomes are the most researched and thoroughly understood subtype of EVs (38). They are characterized by several protein markers, including a cluster of differentiation (CD) (CD63), ALG-2-interacting protein X (Alix), and tumor-susceptibility gene 101 (TSG101) (39). Previous studies have shown that exosomes play a critical part in viral pathogenesis and immunity (40). They assist the host in initiating powerful immune reactions targeting viruses by carrying antiviral substances and activating the antiviral mechanism opposing various viruses in various cells (41).

Hence, in the present study, we hypothesized that CoV alters EV production and content while also controlling the exosome pathway and influencing EV biogenesis and composition in host cells. We assessed the influence of FCoV infection on the biogenesis and composition using CRFK cells produced by EVs. CRFK cells mainly originate from feline kidney cells, which are epithelial classification based on morphology (42). Our results prove that the release of EVs after FCoV infection is time-dependent and leads to an increase in the expression of protein biomarkers. Moreover, EVs were examined for immune response, pathogen progression, and antiviral responses. Our results indicated that EVs derived from FCoV-infected CRFK cells play a crucial role in influencing immune responses. Therefore, EVs could provide diagnostic and therapeutic applications to treat animal CoVs and to obtain insight into the host-cell interactions dealing with FCoV as well as events that may occur when the virus enters the cell. Such knowledge could provide information to prevent future CoV infection in humans and animals.

## 2 Materials and methods

### 2.1 Cell culture

CRFK cells were used in this study as a host model for FCoV infection. They were obtained from the American Type Culture Collection (ATCC). CRFK cells were cultured in Eagle’s Minimum growth media (EMEM) (Fisher Scientific), including L-glutamine supplemented with 10% horse serum (HS) (Fisher Scientific), which is important as a source of growth factors and necessary nutrients needed for cell growth, 1% penicillin/streptomycin (Fisher Scientific), and 0.2% (0.5 μg/mL) amphotericin B (Fisher Scientific). Importantly, exosome-free media was made with 2% exosome-depleted horse serum in compliance with the laboratory procedure for virus infection. CRFK cells were placed in a 37°C incubator enriched with 5% CO_2_ and allowed to reach about 70–80% confluency.

### 2.2 Viral stock

Feline coronavirus [Enteric; Strain: WSU791683 (3)] stock was utilized in this study, which was acquired from ATCC. The viral stock concentration (titer) was 8.9 × 10^6^ TCID_50_/mL. The cytopathic effect (CPE) experiment was performed over a 10-day infection cycle, and the cytopathic effects caused by FCoV on the CRFK cells were determined to obtain the necessary multiplicity of infection (MOI). CRFK cells were exposed to several MOIs (50 IFU, 500 IFU, 2500 IFU, 4000 IFU) of FCoV and noted for CPE at 24-h intervals. CPE describes the alterations in the morphology of host cells following viral intrusion. Examples of these changes include circularizing virus-infected cells and merging with neighboring cells. CPE was not identified with an MOI of 50 IFU and 500 IFU for the duration of 10 days and 8 days after infection, correspondingly. Nevertheless, at a MOI of 2,500 IFU and 4,000 IFU in CRFK cells exhibited CPE following 3 days and 1 day of infection, correspondingly. Therefore, we chose a MOI of 2,500 IFU for further experimentation at 48 h and 72 h incubation time points based on CPE assay.

### 2.3 Infection of CRFK cells

After cell densities reached approximately 70–80% confluency, cells were trypsinized and counted using a countess cell counter. Approximately 5.0 × 10^5^ cells were seeded per cell culture dish and then incubated throughout the night at 37°C and 5% CO_2_. Subsequently, the cell-free medium was removed, and a 2% EMEM medium devoid of exosomes was added per cell culture dish. The EMEM medium was made with exosome-depleted HS, EMEM including L-glutamine, 1% penicillin/streptomycin, and 0.2% (0.5 μg/mL) amphotericin B. Uninfected dishes were used as controls, and infected dishes were infected with feline CoV at a MOI of 2,500 IFU. Both uninfected and FCoV-infected dishes were incubated for 48 h and 72 h time points (37°C and 5% CO_2_). The cell supernatant was separately gathered from FCoV-infected and uninfected dishes and kept at −80°C for later isolation of EVs.

### 2.4 MTT (3-(4, 5-dimethylthiazo-1-2yl)-2,5-diphenyltetrazolium bromide) assay

MTT assay (colorimetric assay) evaluates the cell viability and cytotoxicity that measures metabolic activities. In 96 well plates, 1 × 10^4^ CRFK cells were added independently in triplicates and incubated throughout the night, maintaining 37°C and 5% CO_2_ conditions. The growth medium was discarded on the subsequent day, and a 2% exosome-free medium was added to each well. The following day, the cells were infected with FCoV at MOI of 2,500 IFU, and the infected cells were incubated for 48 h and 72 h while the control wells remained in the exosome-free medium. Cells were exposed to 50 μL of 5 mg/mL MTT in 1X PBS and incubated for a duration of 4 h, maintaining 37°C and 5% CO_2_ conditions. Following incubation, a 100 μL stop solution was introduced to each well. Finally, the absorbance was measured at 570 nm, and every sample was analyzed in triplicates. The number of viable cells was investigated via a bright field microscope and compared to the CRFK cell viability at 48 and 72 h.

### 2.5 Isolation and purification of EVs

Previously, collected control and infected exosome-free cell supernatant were centrifuged at 1,300 Revolutions Per Minute (rpm) for a duration of 10 min at 4°C utilizing an Allegra X-14R Centrifuge. Then, the pellets were discarded, and the supernatant was collected again. Subsequently, the media was subject to centrifugation at 3900 rpm for 10 min using an Allegra X-14R Centrifuge, and then the supernatant was filtered using a 0.22 μm porosity filter. Subsequently, the supernatant was moved into an ultracentrifuge tube, the volume was prepped with 1X PBS and centrifuged at 10,800 rpm for 45 min at 4°C using a Beckman Coulter Optima L-70 K ultracentrifuge. The supernatant was once more gathered, and centrifugation was performed at 32,000 rpm for 70 min at 4°C. The supernatant was removed, and roughly 500 μL of purified EV pellets were saved from each control and infected tube. To inhibit the protein degradation, a protease inhibitor (10 μL/mL) was introduced to the isolated EVs and stored at −80°C until further experimentation (43).

### 2.6 Total DNA/RNA extraction

TRIzol reagent was used for the extraction and purification of DNA and RNA of isolated EVs. EV samples weighing 5 μg were treated with 1 unit (U) of RNase-free DNAase I for DNA extraction and 1 U of micrococcal nuclease (MNase) for RNA extraction. For total DNA, RNase-free DNAase I treated with 5 μg control and infected CRFK-derived isolated EV samples were incubated in a water bath for a duration of 30 min at 37°C. Subsequently, they were processed with 50 mM Ethylenediaminetetraacetic acid (EDTA) treatment for a duration of 10 min at 65°C. DNA isolation was followed through the TRIzol extraction method (44). Total RNA was extracted by incubating EVs with 1% Triton-X-100 on ice for 30 min and exposed to MNase at 37°C for 15 min. Subsequently, the RNA isolation was followed by the TRIzol extraction method. Finally, total DNA and RNA in isolated EV samples were analyzed using Nanodrop (Thermo Scientific).

### 2.7 Bicinchoninic acid (BCA) assay

The quantitation of the total protein of CRFK-derived EVs was analyzed using a BCA assay. Five μL of 0, 0.2, 0.4, 0.8, and 1.6 μg/μL standards [bovine serum albumin (BSA)], CRFK-derived control, and infected EVs were introduced in triplicates in a 96-well plate. Subsequently, BSA protein assay reagents A and B were added, 25 and 200 μL, respectively, to each well. The 96-well tissue culture plate was covered with aluminum foil and positioned on a shaker for a duration of 10 min. Then, the plate was read at 595 nm absorbance. The standard curve was graphed to discover the accurate protein concentration in CRFK-derived control and infected EVs.

### 2.8 NanoSight tracking analysis

NanoSight tracking analysis (NTA) was performed to characterize the nanoparticles as well as to analyze the concentration (particles per mL) and nano-size distribution of the CRFK-derived control and infected EVs based on the rate of Brownian motion and light scattering using a Zeta View R Particle Matrix Tracking Analyzer instrument. For analysis, a 20 μL volume of EV samples was diluted at 1:75 in microbial cell culture-grade water before loading the prepared EV samples into the chamber of the Zeta View instrument. The mean values were investigated at 11 distinct positions.

### 2.9 Scanning electron microscope

Scanning electron microscope (SEM) was performed to examine the surface morphology of the CRFK-derived EVs. The CRFK-isolated EVs were vortexed and stabilized with 2.5% EMS-quality glutaraldehyde at a 1:1 ratio. 30 μL of exosome samples were added in vesicle mixtures to a clean carbon disc-SEM mounting stud. The vesicles were immobilized subsequent to drying and left overnight. Then, the EV samples on a carbon-SEM mounting stud were affixed to an SEM stage utilizing carbon paste. Prior to imaging using Phenom XL G2 Desktop SEM, a 5 nm coating of gold–palladium alloy was implemented by sputtering to improve surface conductivity. SEM was executed within the reduced beam energies. To achieve the best results in vesicle surface morphology under SEM, freshly isolated exosomes were fixed and attached to a conductive, adhesive carbon substrate immediately following isolation and were imaged within a week. An examination of exosome size was performed utilizing the SEM images with the assistance of Image-J software.

### 2.10 Dot blot analysis

Dot blot experiment examines the expression of particular protein markers such as exosomal markers, immune response markers, pathogenic markers, apoptotic proteins, and stress-specific proteins (Hsps) in CRFK-derived EVs. Five μL of CRFK-derived EVs were added to the reducing buffer (1: 1) and were boiled for a duration of 10 min at 95°C. Prepared control and infected EVs were dotted on the nitrocellulose membrane and blocked for 30–45 min with 5% nonfat dry milk to prevent nonspecific bonding at room temperature (RT). Then, the membrane was washed three times for 10 min each with 1× Tris-buffered saline containing Tween-20 (0.2%) buffer solution (TBST) and incubated with primary antibodies including Alix (Fisher Scientific), CD63 (Santa Cruz Biotechnology), TSG101 (Fisher Scientific), ACE2 (DHSB), TMPRSS2 (DHSB), anti-flotillin-1 (BD Bioscience), Clathrin (BD Bioscience), cadherin (DSHB), CD29 (DSHB), anti-TLR3 (Abnova), TLR6 and 7 (Invitrogen), IRF4 (DSHB), mCCL22 (RD Systems), TGFβ-3(DHSB), TNF-α (Bioss Antibodies Inc.), CD47(Bioss Antibodies Inc.), LAMP-1 (human) (DSHB), ATPase (DSHB), TSPAN8, HSPB8-13B6 (Hsp22) (Invitrogen), HSPB1-1 (Hsp27) (DSHB), Hsp100 (DSHB), DIS3-1D7 (DSHB), and cleaved caspase-3 (RD Systems). The nitrocellulose membrane was washed three times on a subsequent day using a TBST buffer solution. Horseradish peroxidase (HRP)-conjugated secondary antibody, goat anti-mouse (Fisher Scientific), goat anti-rat (Fisher Scientific), or goat anti-rabbit (Novus Biologicals LLC) were added to the blocking buffer and incubated with membranes for a duration of 60–120 min. The target protein signals were identified utilizing the Super Signal West Femto Maximum Sensitivity Substrate (Invitrogen). Subsequently, the image was developed through the Bio-Rad ChemiDoc™ XRS+ System (Bio-Rad Laboratories).

### 2.11 Sodium dodecyl sulfate-polyacrylamide gel electrophoresis (SDS-PAGE) and western blot analysis

Protein biomarkers were further analyzed using a western blot. Here, approximately 32 μL of isolated EVs were mixed with reducing buffer at a 1:1 ratio and were boiled (95°C) for a duration of 10 min. Prepared samples were placed in a 4–20% 1.5 mm Bio-Rad precast gel and run at 100 V. The procedure continued overnight until proteins were transferred to the Polyvinylidene difluoride (PVDF) membrane in a transfer chamber at 45 mA. Then, the PVDF membrane was blocked for 35 min using the blocking solution (5% nonfat dry milk) at RT and washed three times for 10 min each using 1× TBS containing Tween-20 (0.2%) buffer solution. Then, the PVDF membrane was incubated with primary antibodies, including ACE2 (DHSB) and TMPRSS2 (DHSB). On a subsequent day, the membrane was washed three times for 10 min with TBST buffer solution. It was incubated with secondary antibodies, which can be either HRP-conjugated goat anti-mouse, goat anti-rat, or goat anti-rabbit, and was added to the blocking buffer for 1–2 h at RT. Finally, signals were developed using Super Signal West Femto Maximum Sensitivity Substrate (Invitrogen).

### 2.12 Statistical analysis

Statistical analysis was conducted using a t-test on the acquired data employing the GraphPad Version 5 software and Bio-Rad imaging program. The statistical significance was calculated by mean value ± standard deviation, and the statistical significance of the *p*-value was described as *p* ≤ 0.05(*), *p* ≤ 0.01(**), *p* ≤ 0.001(***), *p* ≤ 0.0001(****).

## 3 Results

### 3.1 Feline coronavirus altered CRFK cell viability

**Figure 1:**
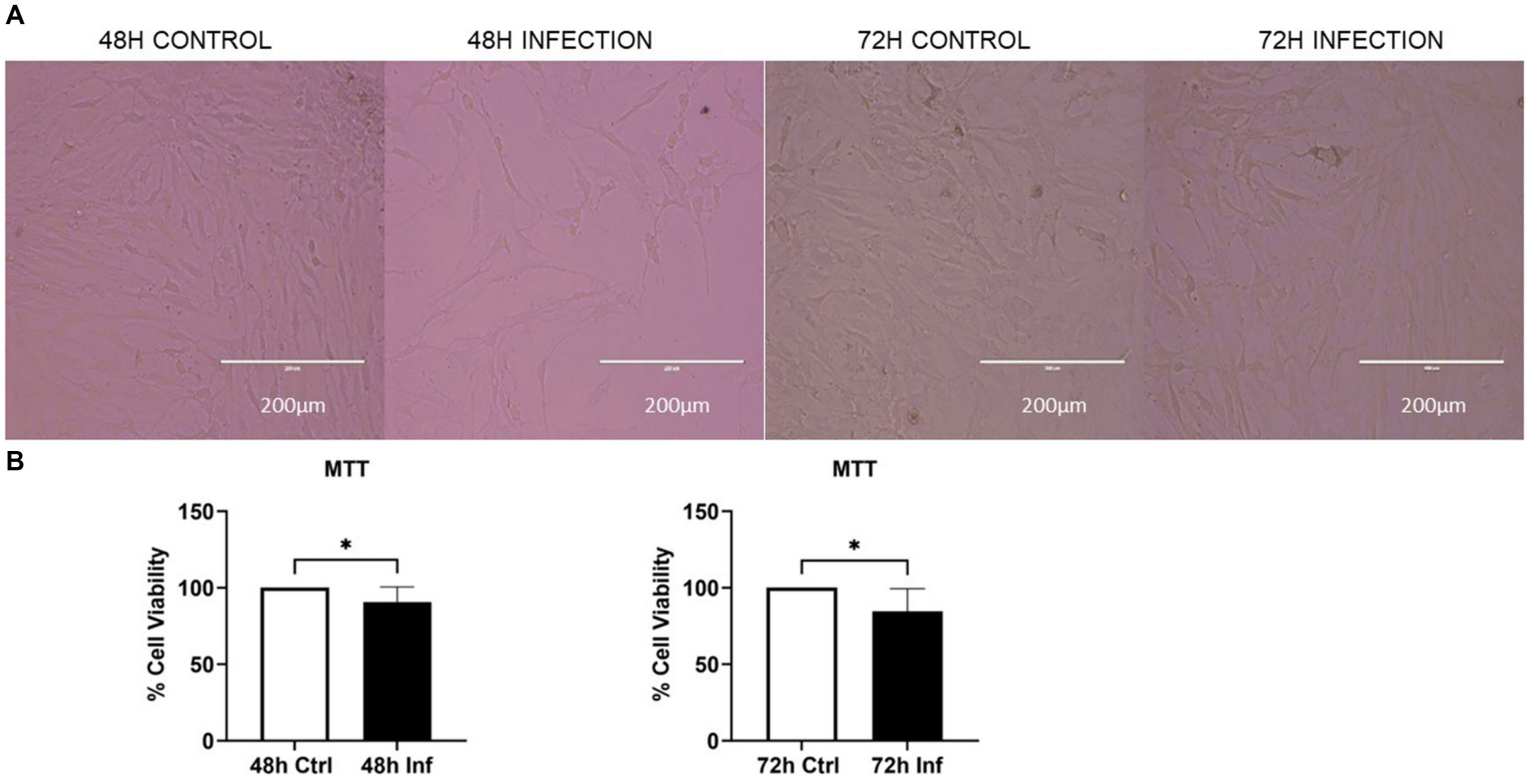
The impact of FCoV on CRFK cell viability. **(A)** Bright-field microscopy images indicate the morphology of CRFK cells at 48 and 72 h. **(B)** CRFK cells were infected with FCoV in exosome-depleted media at a MOI of 2,500 IFU at 48 and 72 h time points. Post-infection enumeration of viable CRFK cells was subsequently incubated with MTT at 37°C for a duration of 3–4 h; absorbance was measured at 570 nm. Statistical analysis of the acquired data points was conducted using a *t*-test. Statistical significance is implied through the mean ± standard deviation (SD) as stated: ∗*p* ≤ 0.05.

The cellular morphology was analyzed using bright-field microscopy, which indicated a reduced CRFK cell count with an increased incubation time after FCoV infection (Figure 1A). The cell viability was assessed utilizing an MTT assay, which exhibited that the cell viability of FCoV-infected CRFK cells was reduced with increased incubation time (Figure 1B). The cell viability of FCoV-infected cells was significantly reduced by nearly 9% (* *p* ≤ 0.05) and 15% (* *p* ≤ 0.05) at 48 h and 72 h, respectively, contrasted to the control CRFK cells. The decrease in cell viability with prolonged incubation times after FCoV infections indicates that FCoV infection significantly induces cell demise in CRFK cells.

### 3.2 Evaluation of CRFK-derived EV size, concentration, surface morphology, and biomolecules

**Figure 2:**
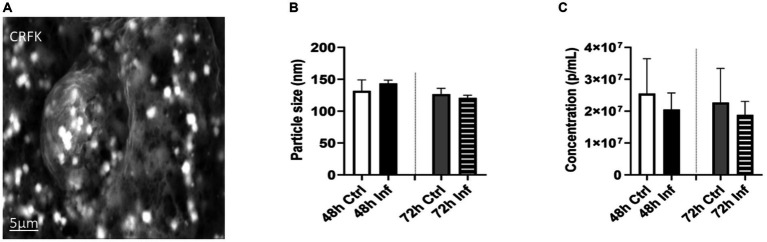
Morphological analysis of FCoV-infected CRFK-derived EVs. **(A)** Scanning electron microscopy (SEM) images demonstrating the surface morphology of the CRFK-derived control EVs at 72 h-time points at 5 μm. **(B)** NTA indicates the CRFK-derived EV’s mean particle size; **(C)** particle concentration after 48 h and 72 h FCoV infection. Statistical analysis of the acquired data points was conducted using a *t*-test.

**Figure 3:**
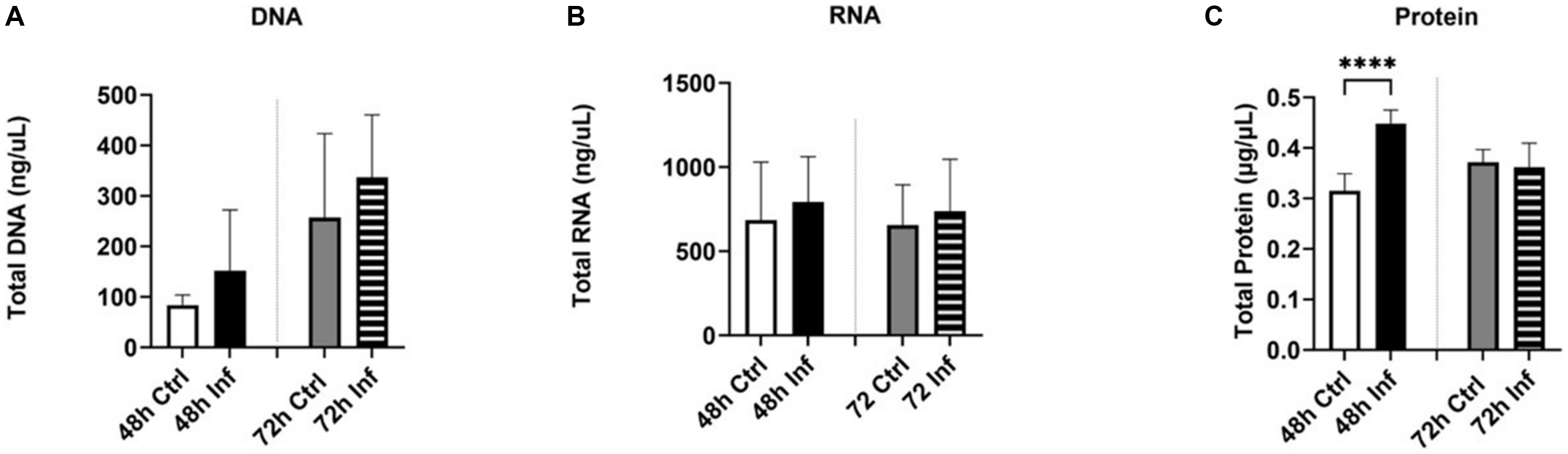
The biological significance of FCoV-infected CRFK-derived EVs. Graphs showing the **(A)** total DNA, **(B)** total RNA, and **(C)** total protein content of CRFK-derived EVs after 48 h and 72 h FCoV infection. Statistical analysis of the acquired data points was conducted using a *t*-test. Statistical significance is implied through the mean ± SD as stated: ∗∗∗∗*p* ≤ 0.0001.

To examine the impact of FCoV on EVs, CRFK cells were infected with FCoV at 48 h and 72 h time points. Cell supernatants were ultracentrifuged through a series of high-speed centrifugation steps to isolate and purify EVs produced from CRFK cells. Isolated EVs were examined using SEM and NTA to identify the surface morphology, particle size in nanometers (nm), and concentration (particles per mL). The SEM image revealed the surface morphology of CRFK-derived control and FCoV-infected EVs in both time references. Figure 2A represents an SEM image, which indicates control CRFK-derived EVs at the 72 h-time point at 5 μm, verifying the presence of EVs in the sample. NTA was performed to characterize EVs by determining the mean particle size (nm) and concentration (particles/mL). The mean particle size was slightly increased (143.4 nm) in FCoV-infected EVs compared to the control EVs (131.9 nm) at 48 h (Figure 2B). The mean particle size was slightly decreased (120.9 nm) in FCoV-infected EVs compared to the control EVs (126.6 nm) at 72 h (Figure 2B). Therefore, NTA analysis confirmed that the average size of EVs ranges from 100 to 200 nm at 48 and 72 h times points. The particle concentration (particles/mL) of FCoV-infected EVs exhibited a negligible decrease compared to the control EVs at both time points (Figure 2C). At 48 h, the mean concentration of control and infected EVs were 2.5 × 10^7^ and 2.0 × 10^7^ particles/mL, respectively. In addition, at 72 h, the mean concentration of control and infected EVs were 2.2 × 10^7^ and 1.8 × 10^7^ particles/mL, respectively. The levels of total DNA, RNA, and protein were analyzed in control and FCoV-derived EVs at 48 h and 72 h times points. Figure 3A indicates that there was a gradually increasing trend in total DNA with increased incubation time. Figure 3B indicates that there was a negligible increase in total RNA levels with increased incubation time. However, the total protein level of FCoV-infected EVs was significantly increased as compared to the control EVs at 48 h (**** *p* ≤ 0.0001) (Figure 3C).

### 3.3 Identification of the presence of classical biomarkers in EVs

**Figure 4:**
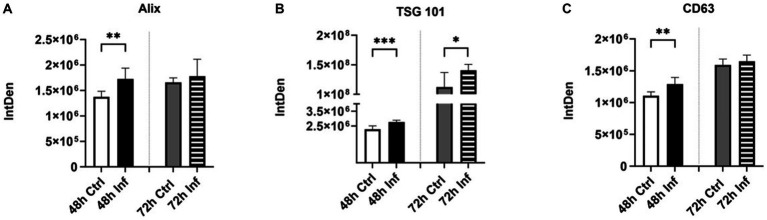
The influence of FCoV infection on CRFK-derived EVs’ classical markers. Graphs indicate the quantitative dot blot analysis of **(A)** Alix at 48 and 72 h (Supplementary Figure S1A); **(B)** TSG101 at 48 and 72 h (Supplementary Figure S1B); and **(C)** CD63 at 48 and 72 h (Supplementary Figure S1C) in CRKF-derived control and FCoV-infected EVs. The dots displayed in the figure indicate the results obtained from six-fold dot blot experiments. Statistical analysis of the acquired data points was conducted using a t-test. Statistical significance is implied through the mean ± SD as stated: ∗*p* ≤ 0.05, ∗∗*p* ≤ 0.01, and ∗∗∗*p* ≤ 0.001.

Classical exosome proteins are used to characterize EVs; they are found on the surface of the exosome membrane and the inner space (45). Alix and TSG101 are multivesicular body (MVB) related proteins engaged in the endosomal-sorting complex, which is necessary for transportation (ESCRT) (45). TSG101 protein marker plays a significant role in the control of growth and differentiation (46). CD63 are tetraspanins that play a crucial function in sorting cargo-like premelanosome protein (PMEL) onto intraluminal vesicles (ILVs) within MVBs (47, 48). In the present investigation, we conducted a dot blot to assess the expression of classical biomarkers. The dot blots of Alix (Supplementary Figure S1A), TSG101 (Supplementary Figure S1B), and CD63 (Supplementary Figure S1C) in control and infected derived EVs were analyzed via the Bio-Rad imaging program and GraphPad. FCoV-infected CRFK-derived EVs presented significantly elevated expression of Alix (∗∗*p* ≤ 0.01), TSG101 (∗∗∗*p* ≤ 0.001), and CD63 (∗∗*p* ≤ 0.01) at 48 h infection and significantly increased expression of TSG101 (∗*p* ≤ 0.05) at 72 h infection relative to the control EVs, respectively, (Figures 4A–C). Our results indicate that FCoV infection affects the abundance of tetraspanins and regulates the ESCRT pathway.

### 3.4 Presence of the host receptors and host cell protease after FCoV infection

**Figure 5:**
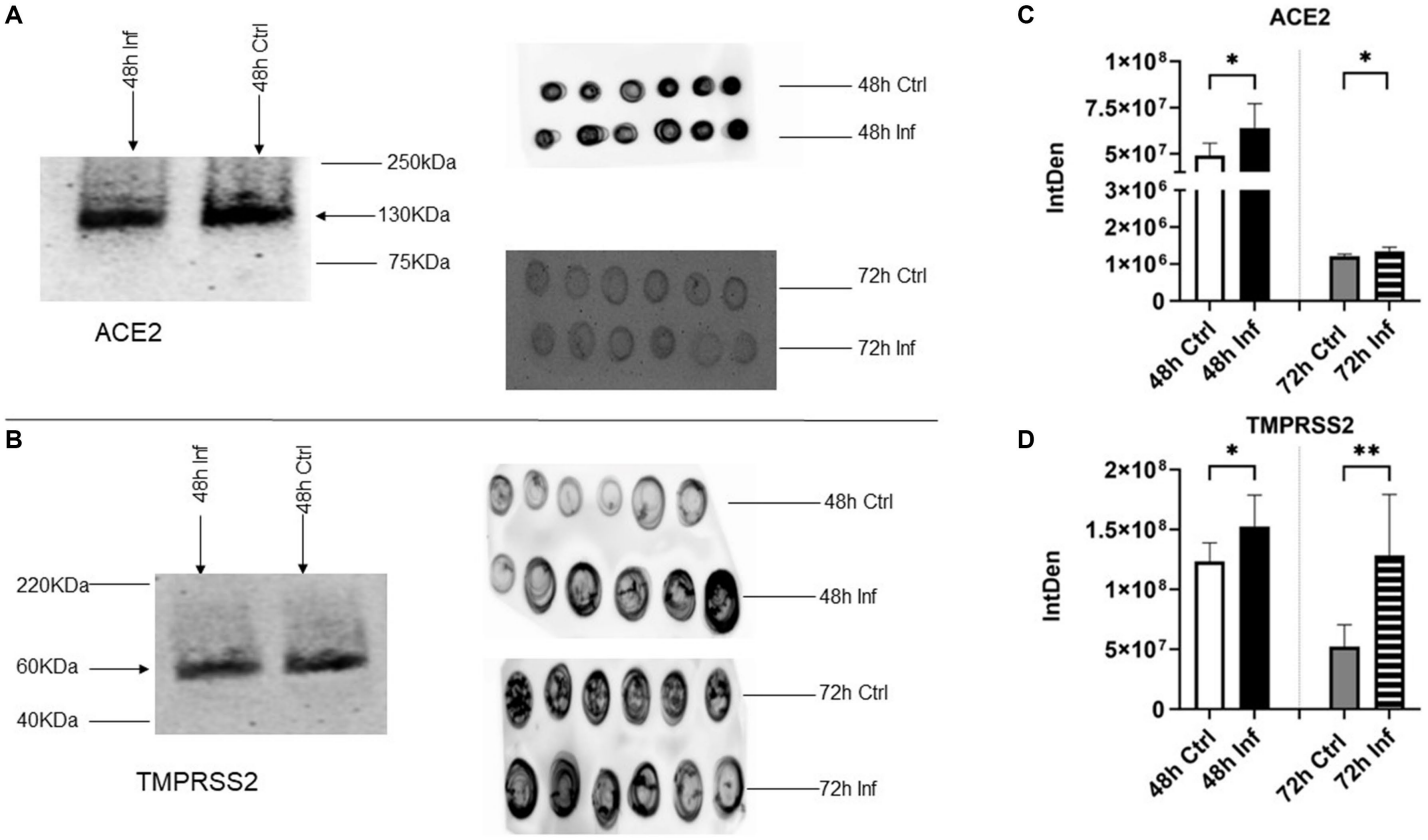
The presence of host receptors and host cell protease in response to FCoV infection. **(A)** Western analysis showing the expression of coronavirus host receptor, ACE2 at 48 h, and dot blot analysis showing the expression of ACE2 at 48 and 72 h in CRFK-derived control and FCoV infected EVs. **(B)** Western analysis showing the expression of CoV host cell protease, TMPRSS2 at 48 h, and dot blot analysis showing the expression of TMPRSS2 at 48 h and 72 h in CRFK-derived control and FCoV-infected EVs. Graphs indicate the quantitative dot blot analysis of **(C)** ACE2 at 48 and 72 h and **(D)** TMPRSS2 at 48 and 72 h in CRKF-derived control and FCoV-infected EVs. The dots displayed in the figure indicate the results obtained from six-fold dot blot experiments. Statistical analysis of the acquired data points was conducted using a *t*-test. Statistical significance is implied through the mean ± SD as stated: ∗*p* ≤ 0.05 and ∗∗*p* ≤ 0.01.

ACE2 has been determined to be a cellular receptor on the cell surface for both SARS-CoV and SARS-CoV-2 (5). ACE2 is a carboxypeptidase, mainly detected in the heart, lungs, and kidneys. It is recognized as a counter-regulator of the renin-angiotensin system (RAS) and substantially influences the cardiovascular system (5, 49). Transmembrane protease, Serine 2 (TMPRSS2), plays a pivotal role as a cofactor in SARS-CoV-2 entry and is able to activate glycoproteins of respiratory viruses (50, 51).TMPRSS2 is found in epithelial cells at different locations, such as respiratory, genitourinary, and gastrointestinal systems (52). ACE2 attaches with the spike protein of SARS-CoV-2 to obtain access to the host cell and serine protease (53). TMPRSS2 is able to activate the S protein and release subunit S2, which facilitates the merging of the viral and cellular membranes. Consequently, viral genes enter the host cell and spread (54, 55). Therefore, in SARS-CoV-2 infection, ACE and serine protease TMPRSS2 play critical roles in viral entry and S protein priming, respectively (54, 56). In our investigation, we evaluated the presence of ACE2 and TMPRSS2 in isolated CRFK-derived control and FCoV-derived EVs and both time points via western blot and dot blot analysis (Figures 5A,B). The western blot analysis of ACE2 and TMPRSS2 indicated gel bands, approximately 130 kilodaltons (kDa) and 60 kDa, respectively. The ACE2 protein marker was significantly increased at 48 h (∗*p* ≤ 0.05) and 72 h (∗*p* ≤ 0.05) in FCoV-derived EVs relative to the control-derived EVs (Figure 5C). TMPRSS2 was found to significantly increase in FCoV infection-derived EVs relative to the uninfected control-derived EVs at 48 h (∗*p* ≤ 0.05) and 72 h (∗∗*p* ≤ 0.01) (Figure 5D). These results indicated the presence of CoV host receptor (ACE2) and TMPRSS2 in the control and FCoV-derived EVs produced from CRFK cells.

### 3.5 Expression of membrane trafficking protein markers following FCoV infection

**Figure 6:**
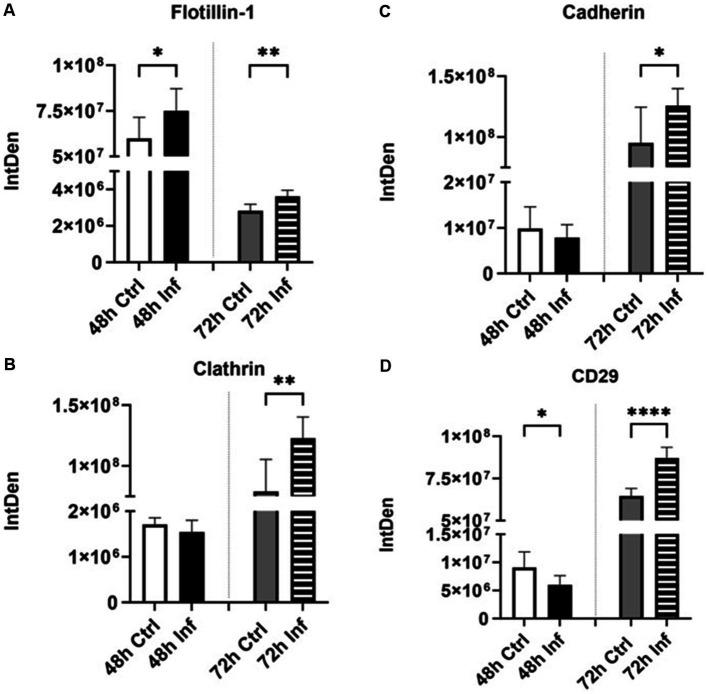
The influence of FCoV infection on membrane trafficking proteins and adhesion molecules. Graphs indicate the quantitative dot blot analysis of **(A)** Flotillin-1 at 48 and 72 h (Supplementary Figure S1D); **(B)** Clathrin at 48 h and 72 h (Supplementary Figure S1E); **(C)** Cadherin at 48 and 72 h (Supplementary Figure S1F); and **(D)** CD29 at 48 and 72 h (Supplementary Figure S1G) in CRKF-derived control and FCoV-infected EVs. Statistical analysis of the acquired data points was conducted using a *t*-test. Statistical significance is implied through the mean ± SD as stated: ∗*p* ≤ 0.05, ∗∗*p* ≤ 0.01, and ∗∗∗∗*p* ≤ 0.0001.

We evaluated the expression of Flotillin-1 and Clathrin membrane trafficking protein markers in isolated CRFK-derived control EVs and FCoV-derived EVs by utilizing a dot blot assay. Flotillin-1 is a membrane-associated lipid raft protein engaged in endocytosis, cell signaling, protein trafficking, protein sorting, and gene expression (57). Levels of Flotillin-1 (Supplementary Figure S1D) in FCoV infection-derived EVs were significantly upregulated at 48 h and 72 h (∗*p* ≤ 0.05 and ∗∗*p* ≤ 0.01, respectively) contrasted with control-derived EVs (Figure 6A). Another membrane trafficking molecule, Clathrin, acts as a prototype self-assembling protein and is able to coat transport vesicles (58). Clathrin is involved in receptor-mediated endocytosis, which plays a significant role in membrane trafficking and mitosis (58, 59). Clathrin expression (Supplementary Figure S1E) in EVs was significantly elevated after FCoV infection at the 72 h (∗∗*p* ≤ 0.01) time point (Figure 6B). Hence, our finding suggested that FCoV infection of CRFK regulates membrane protein trafficking and EV formation.

### 3.6 Expression of adhesion molecules in EV cargoes in response to FCoV infection

The expressions of several adhesion molecules were analyzed in CRFK-derived EV post-infection via dot blot analysis. Cadherin is a transmembrane cell–cell adhesion molecule and plays an important role in tissue morphogenesis by regulating cell signaling (60). Cadherin (Supplementary Figure S1F) was slightly reduced at the 48 h infection time point and significantly increased at 72 h (∗*p* ≤ 0.05) in FCoV-derived EVs relative to the control-derived EVs (Figure 6C). Moreover, we determined the impact of FCoV exposure on CD29 expression (Supplementary Figure S1G and Figure 6D, respectively). CD29 is also known as integrin β-1 (61). Integrins are large proteins composed of α/β-chain subunit cell adhesion molecules and are able to regulate cell adhesion and signaling (62). CD29 was significantly decreased at 48 h (∗*p* ≤ 0.05) and significantly elevated at 72 h (∗∗∗∗*p* ≤ 0.0001) in infected EVs as compared to the uninfected. Therefore, these results indicated that FCoV infection of CRFK cells modulates the process of internalization and encapsulation as well as membrane protein presence within EVs.

### 3.7 FCoV infection stimulated pathogen recognition and proinflammatory responses

**Figure 7:**
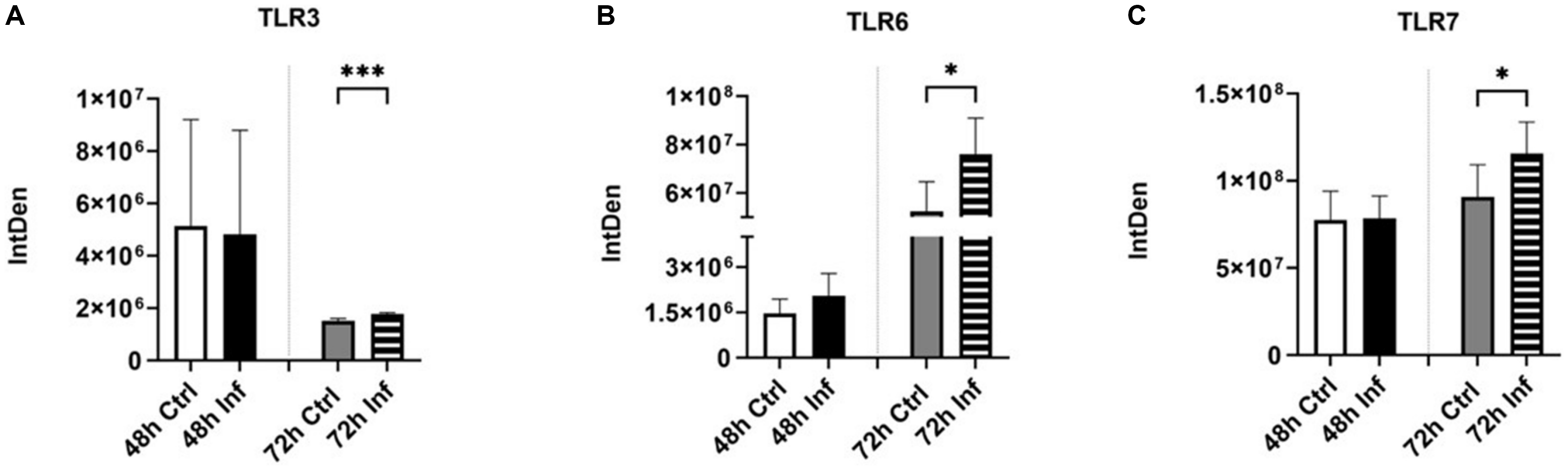
Initiation of pathogen recognition and proinflammatory responses after FCoV infection. Graphs indicate the quantitative dot blot analysis of **(A)** TLR3 at 48 and 72 h (Supplementary Figure S1H); **(B)** TLR6 at 48 and 72 h (Supplementary Figure S1I); and **(C)** TLR7 at 48 and 72 h (Supplementary Figure S1J) in CRKF-derived control and FCoV-infected EVs. Statistical analysis of the acquired data points was conducted using a *t*-test. Statistical significance is implied through the mean ± SD as stated: ∗*p* ≤ 0.05 and ∗∗∗*p* ≤ 0.001.

We evaluated the expression of several toll-like receptors (TLRs) in EVs obtained from CRFK cells after FCoV infection. TLRs are known as pattern recognition receptors, which are accountable for stimulating innate immune responses and pathogen recognition (63). In this study, we investigated TLR3, 6, and 7 expressions via dot blot analysis (Supplementary Figures S1H–J, respectively). TLR3 is a double-stranded RNA (dsRNA), which is found on the endosome membrane and plays a significant role in innate immune responses against viral infections (64). TLR3 is able to activate the transcriptional factors of Interferon Regulatory Factors (IRFs), Nuclear Factor-Kappa β (NF-κβ, and Activating Transcription Factor 1(ATF1) (65). It is also capable of inducing the formation of Interferon-Beta (IFN-β) proinflammatory cytokines (65). We found that TLR3 was significantly increased at 72 h in FCoV-derived EVs relative to the uninfected control EVs (∗∗∗*p* ≤ 0.001) (Figure 7A). Similarly, Figure 7B indicates that the expression of TLR6 was significantly increased at 72 h in FCoV-derived EVs relative to the uninfected EVs (∗*p* ≤ 0.05). TLR2 is capable of forming heterodimers with TLR6, which leads to activating the myeloid differentiation primary response 88 (MyD88)-dependent signaling pathway to induce the production of proinflammatory cytokines (66). TLR6 also activates NF-κB (67). We further analyzed the expression of TLR7, which recognizes single-stranded RNA (SSRNA) viruses (68). It also activates the generation of Tumor Necrosis Factor (TNF) and interleukin-6 (IL-6) proinflammatory cytokines (69). FCoV infection induced a significant upregulation in TLR7 in infected CRFK-derived EVs contrasted with uninfected control EVs at 72 h (∗*p* ≤ 0.05) (Figure 7C). Hence, these findings indicate that TLRs modulate the presence of protein markers associated with proinflammatory and immune activation within the EV’s response to FCoV infection.

### 3.8 Evaluation of protein and inflammatory markers in EVs in response to FCoV infection

**Figure 8:**
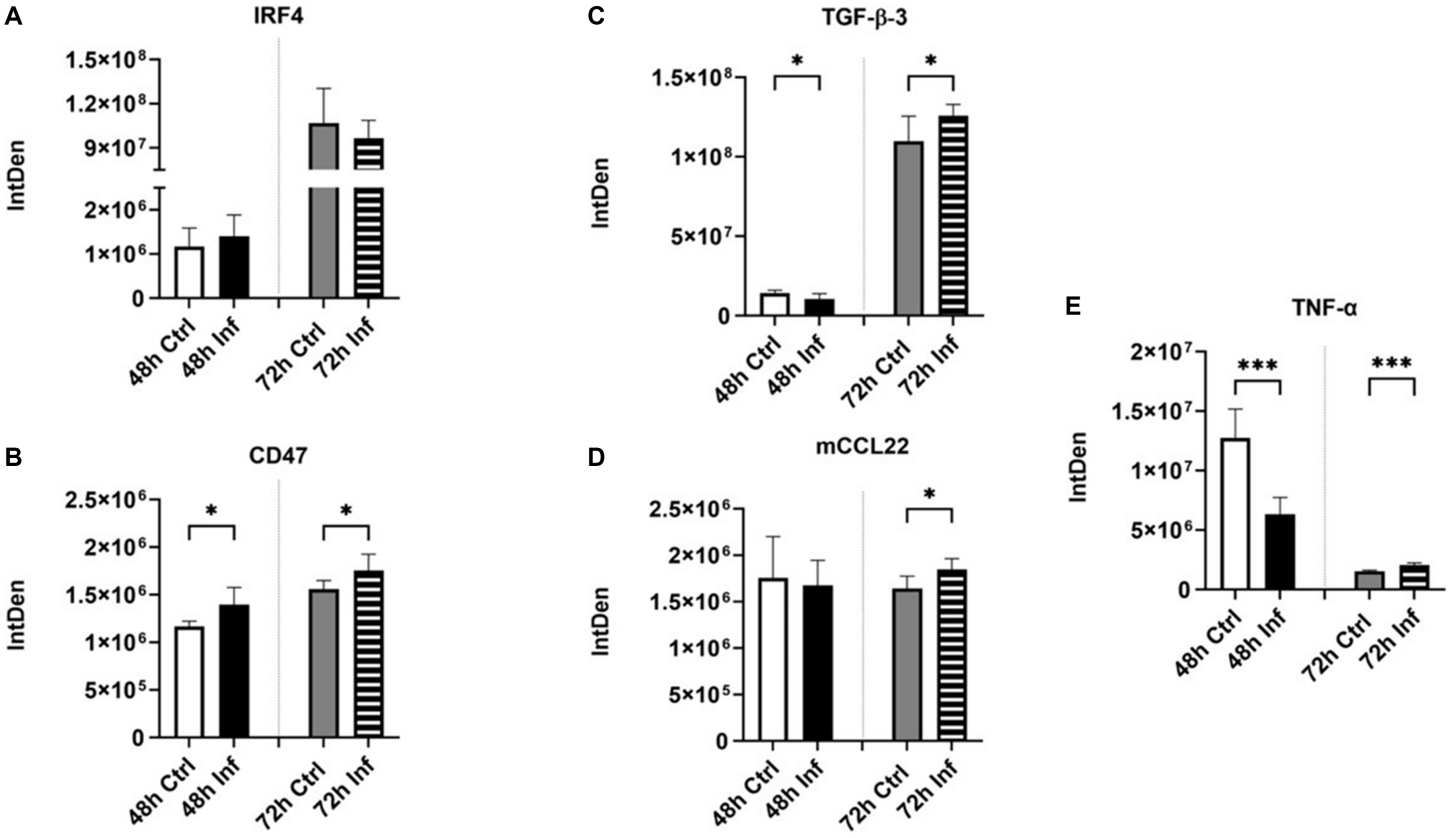
The influence of FCoV infection on CRFK-derived EVs’ protein and inflammatory markers. Graphs indicate the quantitative dot blot analysis of **(A)** IRF4 at 48 and 72 h (Supplementary Figure S1K); **(B)** CD47 at 48 and 72 h (Supplementary Figure S1L); **(C)** TGF-β-3 at 48 and 72 h (Supplementary Figure S2A); **(D)** mCCL22 at 48 and 72 h (Supplementary Figure S2B); and **(E)** TNF-α at 48 and 72 h (Supplementary Figure S2C) in CRKF-derived control and FCoV-infected EVs. Statistical analysis of the acquired data points was conducted using a t-test. Statistical significance is implied through the mean ± SD as stated: ∗*p* ≤ 0.05 and ∗∗∗*p* ≤ 0.001.

We examined the level of immune proteins, including interferon regulatory factor 4 (IRF4) (Supplementary Figure S1K), cluster of differentiation 47 (CD47) (Supplementary Figure S1L), transforming growth factor-beta-3 (TGF-β-3) (Supplementary Figure S2A), and mouse c-c motif chemokine ligand 22 (mCCL22) (Supplementary Figure S2B), and inflammatory markers such as tumor necrosis factor-alpha (TNF-α) (Supplementary Figure S2C) in CRFK-derived EVs after FCoV infection. FCoV infection induced a slight upregulation and downregulation in IRF4 protein levels in FCoV-derived EVs as compared to the control-derived EVs at the 48 h and 72 h time points, respectively (Figure 8A). IRF4 functions as a transcription factor for interferons that play a significant regulatory role in the immune system (70). To further analyze immune proteins, we examined the expression of CD47, a transmembrane immunoglobulin Ig superfamily protein that plays a significant role in inhibiting phagocytosis (71), TGF-β-3 which plays a pivotal role in tissue fibrosis (72), and mCCL22. CD47 was found to have significantly increased in CRFK-derived infected EVs relative to the uninfected control EVs at both 48 h (∗*p* ≤ 0.05) and 72 h (∗*p* ≤ 0.05) (Figure 8B). TGF-β-3 was significantly downreglated in EVs after FCoV infection at 48 h (∗*p* ≤ 0.05) and significantly upregulated 72 h (∗*p* ≤ 0.05) (Figure 8C). Figure 8D indicates that the presence of the mCCL22 protein marker was significantly increased in FCoV-infected EVs contrasted with the uninfected EVs at 72 h (∗*p* ≤ 0.05). We conducted further analysis to examine TNF-α, a cytokine that acts as a key regulator of inflammatory responses (73). Levels of TNF-α were significantly lower in CRFK-derived infected EVs relative to the uninfected control EVs at 48 h (∗∗∗*p* ≤ 0.001) and significantly higher at the 72 h (∗∗∗*p* ≤ 0.001) than that in the control-derived EVs at 72 h time point (Figure 8E). Therefore, these results demonstrated the expression of immune and inflammatory response-associated biomarkers in EVs obtained from CRFK cells following FCoV infection.

### 3.9 Transmembrane molecules, activation of stress-specific and apoptotic response markers expression in response to FCoV infection

We investigated the expression of several transmembrane molecules, stress-specific heat shock proteins (Hsps), and varying caspases in control and infection-derived EVs after FCoV infection. We measured the expression of LAMP-1 (human) (Supplementary Figure S2D), a transmembrane glycoprotein that can be found in lysosomes (74), ATPase (Supplementary Figure S2E), and TSPAN8 (Supplementary Figure S2F) transmembrane protein in CRFK-derived EVs’ post-infection. We detected that LAMP-1 was significantly increased at 48 h and 72 h, respectively (∗*p* ≤ 0.05 and ∗*p* ≤ 0.05). ATPase was significantly upregulated at 72 h (∗*p* ≤ 0.05) after FCoV infection (Table 1). Additionally, TSPAN8 was significantly downregulated and significantly upregulated at the 48 h (∗*p* ≤ 0.05) and 72 h (∗*p* ≤ 0.05) time points, respectively (Table 1) in FCoV-derived EVs when compared to the control-derived EVs. Therefore, these results indicated that FCoV infection modulates the transmembrane protein expression in CRFK-derived EVs and the packaging of EVs.

**Table 1:** The influence of FCoV infection on transmembrane molecules, caspases, and initiation of stress-specific responses in response to FCoV infection.

**Category**	**Protein Makers**	**48 h**	**72 h**
Transmembrane molecules	LAMP-1 (Human)	Significantly Upregulated	Significantly Upregulated
	ATPase	Slightly Upregulated	Significantly Upregulated
	TSPAN8	Significantly Downregulated	Significantly Upregulated
Activation of stress-specific responses after FCoV infection	HSP22	Significantly Downregulated	Significantly Upregulated
	HSP100	Significantly Upregulated	Significantly Upregulated
	HSP27	Slightly Downregulated	Slightly Upregulated
	DIS3	Significantly Upregulated	Significantly Upregulated
Activation of apoptotic responses to FCoV infection	Cleaved Caspase-3	Significantly Upregulated	Significantly Upregulated

Table indicates the result of quantitative dot blot analysis of LAMP-1 (human) (Supplementary Figure S2D), ATPase (Supplementary Figure S2E), TSPAN8 (Supplementary Figure S2F), HSP22 (Supplementary Figure S2G), HSP100 (Supplementary Figure S2H), HSP27 (Supplementary Figure S2I), DIS3 (Supplementary Figure S2J) and cleaved caspase-3 (Supplementary Figure S2K). Statistical analysis of the acquired data points was conducted using a *t*-test. Statistical significance is implied through the mean ± SD as stated: ∗*p* ≤ 0.05 and ∗∗∗*p* ≤ 0.001.

Hsps are recognized as molecular chaperones involved in unfolding cellular proteins due to stress or high-temperature (75). We analyzed the FCoV-infected EVs for the presence of Hsp22, Hsp100, Hsp27, and DIS3 (Supplementary Figures S2G–J, respectively) (Table 1). At the 48 h infection time point, Hsp22, Hsp100, Hsp27, and DIS3 were confirmed to be significantly downregulated (∗*p* ≤ 0.05), significantly upregulated (∗*p* ≤ 0.05), slightly downregulated, and significantly upregulated (∗*p* ≤ 0.05), respectively in FCoV-derived EVs in contrast with the uninfected control EVs. Additionally, at the 72 h time point, Hsp22 (∗*p* ≤ 0.05), Hsp100 (∗*p* ≤ 0.05), and DIS3 (∗∗∗*p* ≤ 0.001) were significantly upregulated, and Hsp27 was slightly upregulated after FCoV infection. These findings confirmed that these stress-specific protein markers were regulated during FCoV infection.

Caspases play a significant role in cell death and inflammation responses (76). The expression of cleaved caspase-3 (Supplementary Figure S2K) was significantly increased at 48 h (∗*p* ≤ 0.05) and 72 h (∗∗∗*p* ≤ 0.001) in FCoV-derived EVs relative to the uninfected control EVs (Table 1). Hence, our results show that FCoV infection influences the caspase protein in EVs produced by CRFK cells.

## 4 Discussion

Virus-infected cells are able to release EVs and facilitate the spread of virus infection (77). EVs play a significant role in viral entry, spread, and immune responses against viral infections and stimulating antiviral mechanisms (22, 77). During viral infections, EVs can assist in transferring viral genomes into specific cells and are involved in cell physiology to aid the infection (22). Delivering drugs to target cells is one of the key issues in pharmacology, and adding specific targets onto the EV membrane might assist in delivering drugs to specific cells (78, 79). Studies have reported that in COVID-19, EVs facilitate the development of a prothrombotic state, which leads to vascular impairment and heightened hypertyrosinemia and disrupts both innate and adaptive immune responses (80, 81). However, there is still a lack of studies on CoVs. In recent years, more studies have focused on the exosomes released from the mesenchymal stem cells as a promising treatment for COVID-19 infection (82).

In our study, we investigated how FCoV infection modulated CRFK-derived EV production, content, biogenesis, and composition. The CRFK cells were infected with FCoV at an MOI of 2,500 IFU and incubated at 48 h and 72 h. Examination through MTT assay revealed that CRFK cell viability was significantly reduced with increased incubation time after FCoV infection (Figure 1B). These results indicated that FCoV infection with increased incubation time leads to upgraded CRFK cell’s cytotoxic activity, confirming a decreased CRFK cell survival rate. Morphological characteristics, including particle size, particle concentration, surface morphology, total DNA, RNA, and protein levels, were examined in CRFK-derived uninfected and FCoV-infected EVs. Moreover, Figure 3A indicates a gradually increasing trend in total exosomal DNA, and Figure 3B indicates a negligible increase in total exosomal RNA levels as time progressed. The total protein content (Figure 3C) was significantly increased at 48 h in FCoV-infected EVs relative to the control EVs, suggesting that FCoV infection elevated EV production, assembly, and release from CRFK cells.

In this study, we investigated the expression of several protein markers, including classical biomarkers (Alix, TSG101, CD63), membrane trafficking molecules (flotillin, clathrin), adhesion molecules (cadherin, CD29), virus-specific host receptor/protease markers (ACE2, TMPRSS2), pathogen recognition markers (TLRs), immune biomarkers (IRF4, TNFα, mCCL22, CD47, TGF-β-3), transmembrane markers (LAMP-1 (human), ATPase, TSPAN8), stress response markers (Hsps, DIS3), and apoptotic response markers (cleaved-caspase-3) in CRFK-derived control and FCoV-infected EVs at 48 h and 72 h time points. We discovered that classical exosome markers Alix (Figure 4A), TSG101 (Figure 4B), and CD63 (Figure 4C) levels were significantly upregulated in FCoV-infected EVs relative to the control EVs. Alix and TSG101 are MVB-related proteins that engage in the endosomal-sorting complex, which is essential for transport (ESCRT) (45, 46). CD63 are tetraspanins that serve a key function in understanding cargo sorting, such as PMEL, into intraluminal vesicles and biogenesis within EVs (47, 83, 84). Hence, the FCoV infection may stimulate the generation of intraluminal vesicles, modulate the ESCRT pathway, and enhance EV release. Another tetraspanin-associated marker is CD29 (Integrin-β-1), which plays a crucial role in cell adhesion, organ development, signal transduction, and tissue repair (62, 85, 86). Moreover, the integrin–tetraspanin complex can be mediated by the exosome uptake (87), and the tetraspanin-rich exosomal membrane might enhance the SARS-CoV-2 internalization and cellular penetration (88). CD29 was significantly upregulated in infected EVs, which could suggest higher FCoV particle entry, modulated internalization, encapsulation, and membrane protein expression in EVs (Figure 6D). Additionally, cadherin, a transmembrane cell–cell adhesion molecule that plays an important role in tissue morphogenesis, was significantly increased in the EVs during FCoV infection (Figure 6C) (60). Flotillin-1 (Figure 6A) and clathrin (Figure 6B) membrane trafficking protein markers were significantly elevated in FCoV-derived EVs relative to the control EVs. Flotillin-1 is a membrane-associated lipid raft protein mainly involved in endocytosis, cell signaling, protein trafficking, protein sorting, and gene expression (57). The significantly increased level of flotillin-1 in the infected EVs induced an increase in endosomal sorting, EV release, and elevated protein recruitment within the lipid raft. Clathrin plays a key role in receptor-mediated endocytosis, which is essential to membrane trafficking and mitosis (58, 59), and clathrin-coated vesicles are involved in EV release and uptake (89).

Literature sources have documented that ACE2 and TMPRSS2 have been involved in SARS-CoV-2 (56). ACE2 functions in the capacity of the primary entry receptor of SARS-CoV-2 (90), and TMPRSS2 is able to activate SARS to facilitate both virus-cell and cell–cell fusion (91). Research studies highlighted the transfer of ACE2 by EVs among different cells, confirming the capability for SARS-CoV-2 to associate with ACE2 on EVs (92). This finding elicited the concept of inhibiting EV trafficking as an antiviral strategy against SARS-CoV-2 infection (92). In addition, other studies discovered that EVs play an important role in transmitting CoVs and producing EVs within the host when EVs have packaged and expressed ACE2 at markedly elevated levels (92). CRFK-derived EVs at the 48 h and 72 h infection time points contained significant levels of ACE2 (Figure 5C) and TMPRSS2 (Figure 5D), which may confirm that infected EVs serve an important function in assisting the dissemination of CoV and extracellular virus production in the host. The role of these protein markers in viral entry affects EV biogenesis and composition. TLR3 (Figure 7A), TLR6 (Figure 7B), and TLR7 (Figure 7C) significantly increased at 72 h in FCoV-derived EVs compared to the control-derived EVs. TLRs are pattern-recognition receptors that are vital in stimulating innate immune responses and pathogen recognition (63). They are located on immune cells, including macrophages, dendritic cells, neutrophils, mast cells, and natural killer cells (63). TLR3 participates in the activation of the transcriptional factors of IRFs, NF-κB, and Activating Transcription Factor 1(ATF1). TLR3 triggers the formation of IFN-β and proinflammatory cytokines (65). TLR6 contributes to activating myeloid differentiation primary response 88 (MyD88) (66, 67), and TLR7 triggers the formation of TNF and IL-6 proinflammatory cytokines (69). Therefore, our finding confirmed that the presence of TLRs provides a defensive mechanism against FCoV infection by controlling the expression of inflammation and immune response-associated markers. Furthermore, CD47 (Figure 8B), TGF-β-3 (Figure 8C), mCCL22 (Figure 8D), and TNF-α (Figure 8E) immune biomarkers were significantly increased in infected EVs relative to uninfected control EVs. While IRF4 (Figure 8A) was expressed, it was significantly unchanged in isolated EVs.

CD47 engages in inhibiting phagocytosis (71). Literature has reported that there is a relationship between EV secretion and TGF-β-triggered inflammatory changes (93). TNF-α is a key regulator of inflammatory responses (73). Hence, these results confirmed that FCoV infection has the ability to control the immune responses within CRFK-derived EVs, which helps to understand the host-virus interaction and host immune responses to the FCoV. Expression of several transmembrane molecules such as LAMP-1 (human), ATPase, and TSPAN8 transmembrane proteins was significantly upregulated in CRFK cell-derived EVs’ post-infection. Transmembrane molecules play a significant role in budding and releasing viruses, including CoV infection. Detection of Hsps, such as Hsp22 and Hsp100, were significantly increased at 72 h post-infection, and DIS3 was revealed to significantly increase at 48 h and 72 h in EVs post-infection (Table 1). Hsps mainly act as molecular chaperones. They are able to protect damaged proteins from heat and unfold aggregated proteins (94). Hsps play an important role in identifying virus entry, replication, and survival during host-viral interaction (95). Hence, these results can provide insight into all viral infections, including CoV infection. Furthermore, we examined stress-specific biomarkers; cleaved caspase-3 was significantly elevated at different time points after FCoV infection (Table 1), which indicated inflammation and severe disease. Caspases are mainly engaged in cell death and inflammation responses (76). Caspases are involved and active in SARS-CoV-2 infection (96). Furthermore, there was an increase in active caspase-3 in SARS-CoV-2 infected patients’ cortical organoids and glial cells, which indicates a solid association between SARS-CoV-2 and initiation of apoptosis (96). Hence, our results confirmed that EVs released from FCoV-infected CRFK cells stimulate apoptotic pathways mediated by cleaved caspase-3 and are able to facilitate the CoV-induced inflammatory responses. These findings of key biomarkers, such as stress-specific proteins, immune response-specific proteins, and apoptosis proteins in the context of FCoV infection, provide new prospects for focused therapeutic interventions. For example, regulating Hsp100 activity can hinder the process associated with viral replication, and the function of caspase proteins in regulating cell death and inflammation emphasizes the promise of apoptosis protein inhibitors to mitigate tissue injuries and enhance therapeutic outcomes (76, 95, 97, 98). Furthermore, focusing CD47 on improving immunological cell detection and eliminating diseased cells could enhance the host organism against viruses’ immune defenses (99–101). Hence, it is important to comprehend the interaction between these protein markers and various immunological routes, which culminates in the evolvement of in-depth and efficient treatment regimens.

## 5 Conclusion

EVs play significant roles during viral infection, including facilitating cell-to-cell communication, transporting viral genetic materials into specific cells, as well as being involved in cell physiology and virus entry that aids viral infection. Hence, EV-based technology demonstrates great potential for disease diagnosis and therapeutic tools to prevent CoV and other viral infections. Our study has confirmed that *in vitro* FCoV has a significant effect on CRFK cell viability and survival, which supports the fact that FCoV-infected CRFK-derived EVs are able to introduce stress responses and apoptotic pathway signals. We found that FCoV-infected CRFK-derived EVs trigger EV production in response to the infectious agent. This study revealed that under the stress induced by FCoV infection, CRFK-derived EVs can efficiently package markers and proteins, providing a clear indication of the impact of the virus on the physiological state of feline cells.

Future studies will investigate the receptor-agnostic penetration of CoV into additional target cells (e.g., lung, spleen, and gastrointestinal) and the synthesis of extracellular vesicles facilitated by the host. Furthermore, gene expression studies need to be investigated to explore the varied specific gene functions and modulation mechanisms of post-CoV infection. However, conducting more in-depth investigations of the interspecies transition of animal CoVs and their adaptation to human carriers is crucial. Hence, it is necessary for further exploration to study virus-host interaction in various animals associated with close contact with humans to prevent future CoV strain mutation. The result of this research could improve the insight into the EVs’ engagement in viral infections and the significance of the therapeutic utility of EVs.

## Data availability statement

The original contributions presented in the study are included in the article/Supplementary material, further inquiries can be directed to the corresponding author/s.

## Ethics statement

Ethical approval was not required for the studies on animals in accordance with the local legislation and institutional requirements because only commercially available established cell lines were used.

## Author contributions

SW: Methodology, Validation, Writing – original draft, Writing – review & editing, Formal analysis, Investigation. RP: Supervision, Writing – review & editing. AI: Writing – review & editing. BC: Writing – review & editing. QM: Conceptualization, Formal analysis, Methodology, Project administration, Validation, Writing – original draft, Writing – review & editing.
