# Supplementary figures and images for: Feline coronavirus influences the biogenesis and composition of extracellular vesicles derived from CRFK cells
Source: Front Vet Sci. 2024 Jul 18;11:1388438. doi: 10.3389/fvets.2024.1388438 (PMC11292801; doi:10.3389/fvets.2024.1388438)

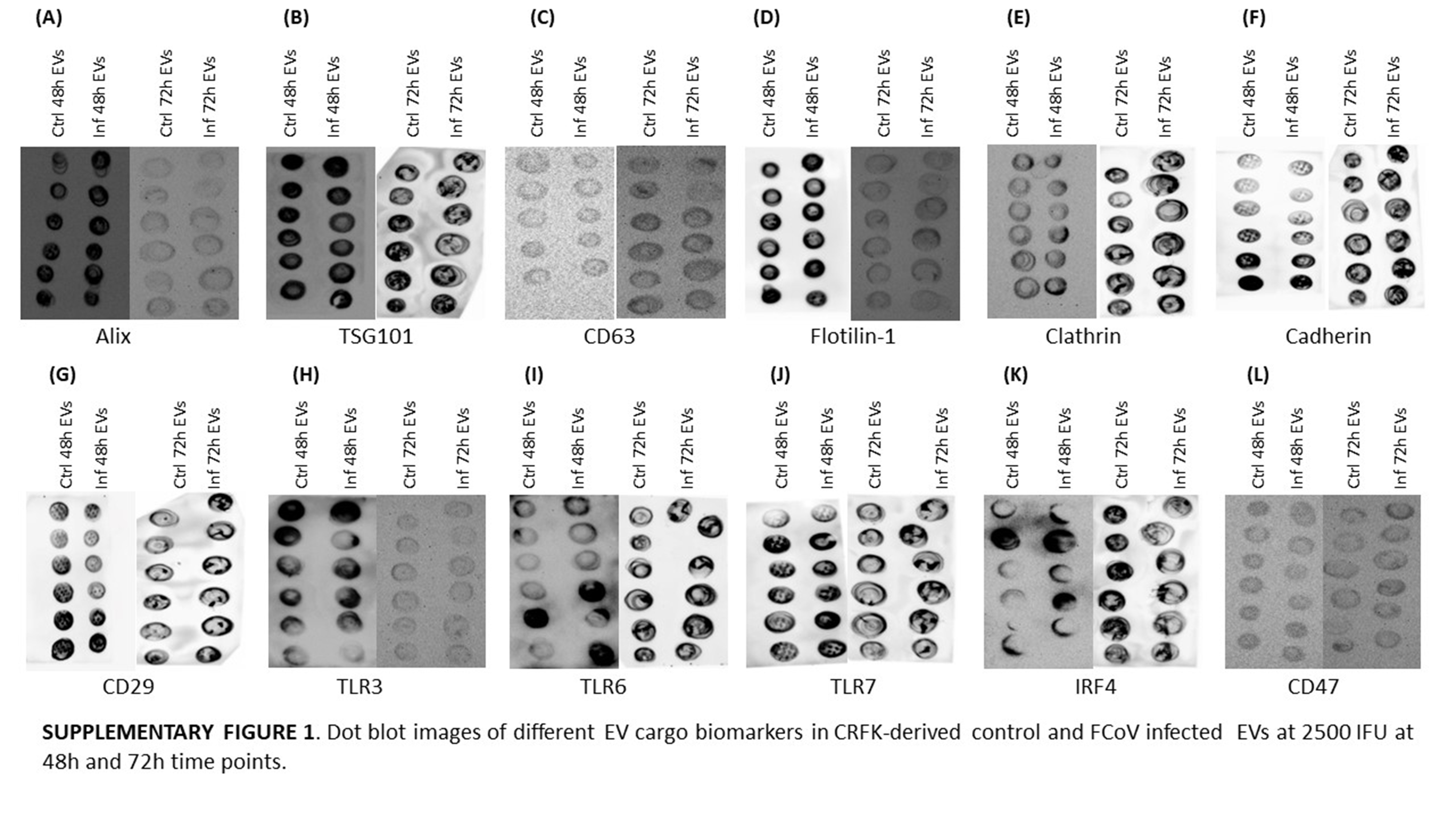

Supplement: Supplementary file 1 [file Image_1.JPEG]

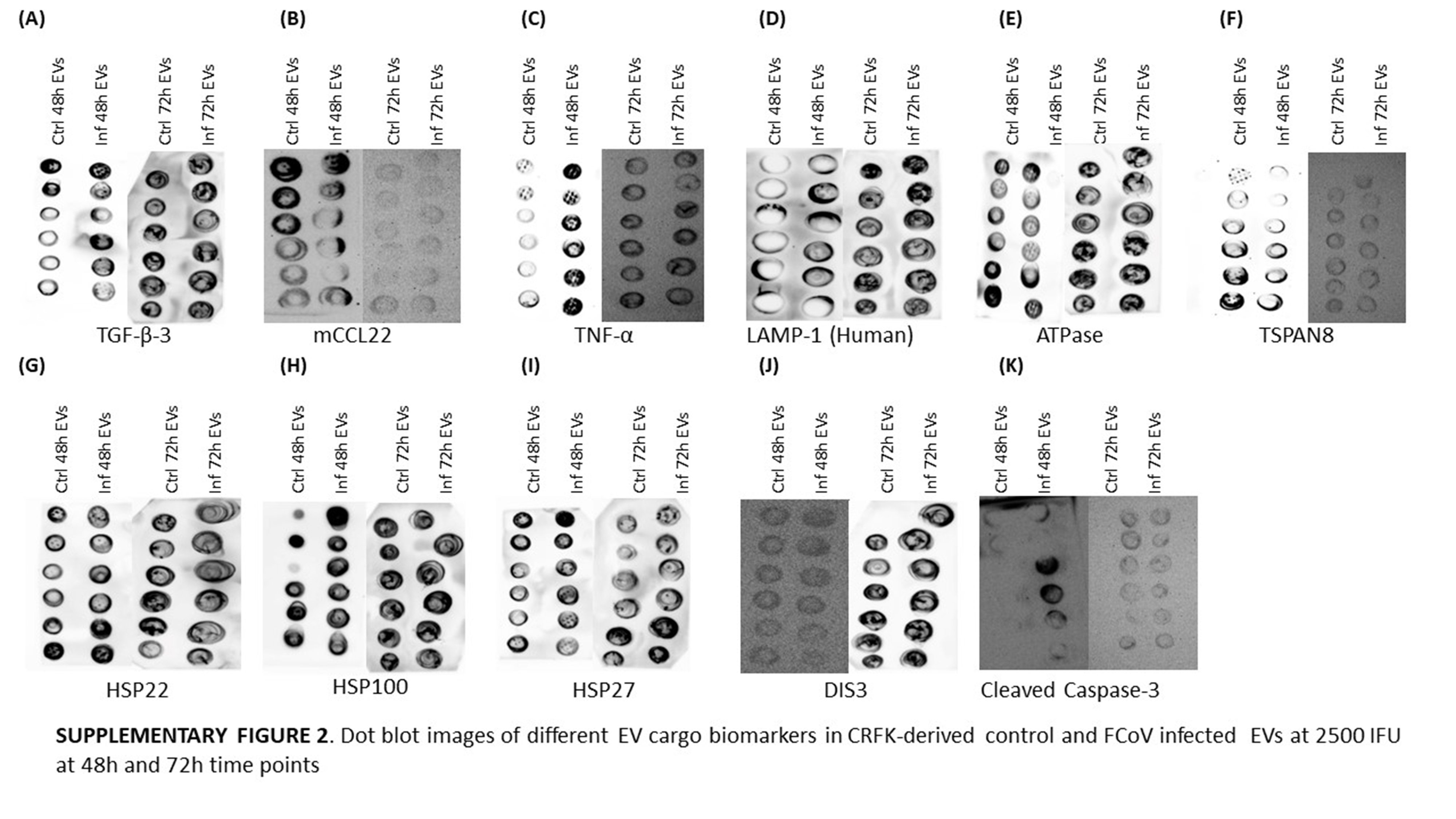

Supplement: Supplementary file 2 [file Image_2.JPEG]
